# The Role of Hyaluronan in Innate Defense Responses of the Intestine

**DOI:** 10.1155/2015/481301

**Published:** 2015-03-30

**Authors:** Carol A. de la Motte, Sean P. Kessler

**Affiliations:** Department of Pathobiology, Lerner Research Institute, Cleveland Clinic, 9500 Euclid Avenue, Cleveland, OH 44195, USA

## Abstract

Hyaluronan is an abundant extracellular matrix component prevalent in the vertebrate intestinal tract. Here we discuss what is known about hyaluronan distribution during homeostasis and inflammatory diseases of the gut and discuss ways in which this glycosaminoglycan can participate in regulating innate host defense mechanisms. These natural responses include mechanisms promoting rapid leukocyte recruitment after bacterial challenge/colon tissue damage as well as promoting epithelial defense mechanisms in the intestine.

## 1. Introduction

Hyaluronan is an abundant extracellular matrix component prevalent in the vertebrate intestinal tract [1, 2], as it is in most organs in the body. HA consists exclusively of repeating disaccharides of N-acetyl glucosamine and glucuronic acid in a nonbranched, linear structure. Uniquely, this glycosaminoglycan does not have a protein core and does not naturally carry additional chemical modifications [3], such as sulfate or nitrate groups. In normal tissue, HA is present as large size polymers (typical range between 10^6^ and 10^7^ Da or from 2500 to 25000 disaccharide units) where it plays a role in tissue hydration, structure maintenance, and elasticity. HA interaction with water is critical for the polymer's biophysical properties, as was appreciated many years ago and reviewed by Comper and Laurent in 1978 [4]. A recent estimate suggests that 15 water molecules can associate with each HA disaccharide of a polymer in a hydration layer [5]. Additionally, due to the fact that HA is mostly present as uniquely large size polymers in the extracellular matrix of healthy tissue, the flexible HA macromolecules form hydrodynamic domains that occupy large water volumes, which inhibit penetration of protein molecules while allowing free diffusion of small molecules [4]. Intrachain covalent bonds between the sugars somewhat restrict HA polymer flexibility, which promotes the formation of larger hydrodynamic domains than a random coil structure would occupy. This HA domain structure directly contributes to the stiffness and flexibility of tissues [4].

## 2. Discussion

### 2.1. The Role of HA in Normal Intestinal Physiology

**Figure 1:**
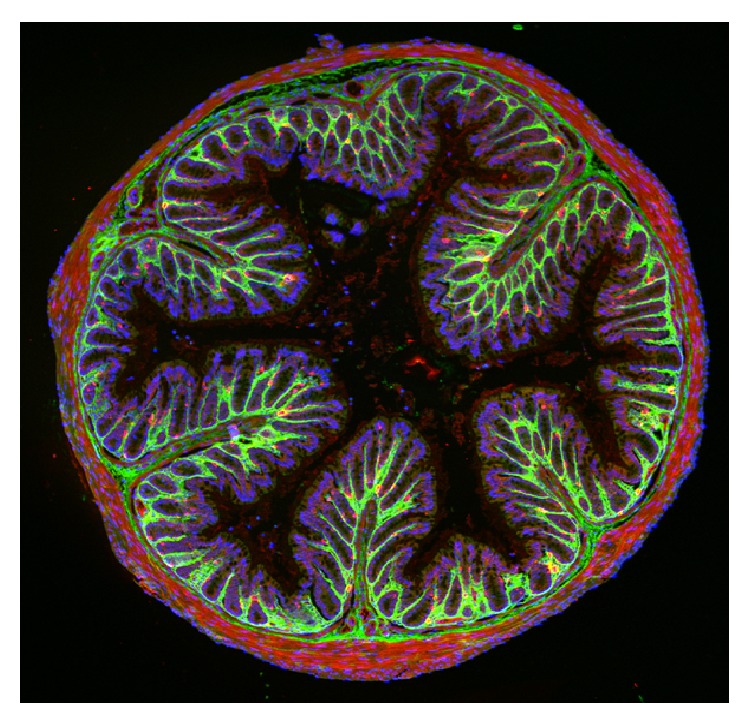
Hyaluronan is a normal component of the large intestine. Cross-section of the distal colon from a healthy mouse, fixed and stained for HA [8] (using biotinylated HA binding protein and a fluorescent detection reagent, green), shows normal deposition around the epithelial-lined crypts. Muscle cells are stained red and cell nuclei are stained blue.

The intestine plays numerous specialized roles in the body, the best known of which are those of water and nutrient absorption and HA likely facilitates these functions through interactions with water. Of the average 9 L of fluid presented to the human intestine daily, 80% is absorbed by the small intestine, 18% is absorbed by the colon, and only 2% is not absorbed [6] (Granger). The lymphatic and blood microvessels control water and solute transport, through continuous interaction with glycosaminoglycan-collagen rich ECM of the interstitium. The dynamic matrix, specifically the hyaluronan component, ensnares water and regulates fluid exchange to and from the blood [6]. Göransson et al. demonstrated in rodent models that HA content in the colon, is up to four times higher than in small intestine [7]. However, HA levels do not change in the colon with water loading, unlike kidney levels of HA, which are loading dependent. Indeed, HA is prominently located immediately beneath the barrier epithelium of the gut (Figure 1) in both healthy humans [8] and mice [9]. Interestingly, during pathologic conditions, such as colitis, the distribution of HA is severely altered [8, 9] at the same time in which water and nutrient uptake are impaired, suggesting a causal connection, although concrete data are scarce.

**Figure 2:**
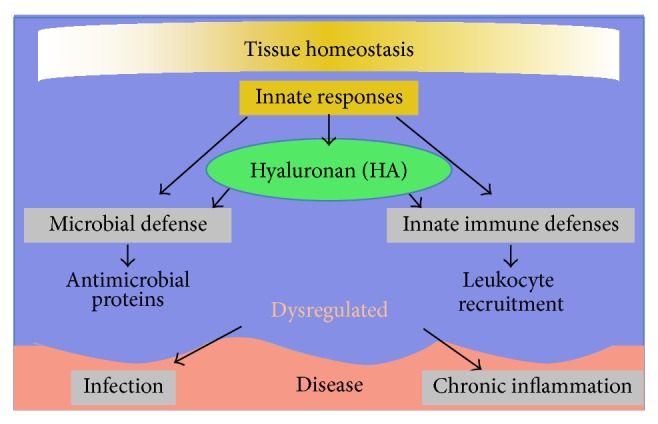
HA plays roles in normal intestinal tissue homeostasis and functions in innate immune and antimicrobial responses. Dysregulation of HA-mediated responses may contribute to chronic inflammation or insufficient protection against intestinal infection.

An additional function of the intestine, one frequently overlooked, is that of playing host to the majority of the microbiome or the collection of beneficial, symbiotic microorganisms that live at especially high concentrations in the large intestine (colon). An estimated 2.5 kg of bacteria make up the microbiome in an adult human [10], and it is the intestine's responsibility to foster the bulk of this beneficial microorganism population while still excluding them from the sterile space within the body. As all body surfaces in contact with the nonsterile environment, the epithelium accomplishes this function. When the intestinal barrier is attacked by invading pathogens (viruses and bacteria) or when colonizing microorganisms cross the epithelial border as a consequence of intestinal damage or disease, rapid innate responses by epithelium and the immune system are critical for defense. HA is now recognized to have important functions in multiple innate host defense mechanisms [11–15] (Figure 2), and dysregulation of production and/or breakdown of HA may promote intestinal disease in ways discussed further below.

### 2.2. HA in Intestinal Inflammation

Far from being merely a static structural component, HA is a dynamic substance that can participate in innate immune responses of the intestine. In cell culture models, it has been known for some time that cytokine-activated leukocytes, that is, blood cells already involved in an immune response, can bind to HA via the cell surface receptor, CD44 [16, 17]. More than 15 years ago, direct binding of nonactivated mononuclear leukocytes to HA produced by virally activated intestinal mesenchymal cells was also demonstrated [18], and this data suggested that HA could also function in leukocyte retention and/or recruitment in the intestinal extravascular space during intestinal disease/damage. Importantly, in patients with chronic intestinal inflammation, as happens in Crohn's disease and ulcerative colitis (two major forms of inflammatory bowel disease), HA accumulates in the colon in the nonvascular space and is in intimate contact with the infiltrating leukocytes [8]. This finding is consistent with many reports associating increased HA deposition in other organs, such as the liver, kidney, and lung (reviewed recently by Jiang et al. [12]) when undergoing inflammation.

HA, as mentioned, is an abundant matrix component within the colon and does not ordinarily promote inflammation. This raises the question of what modifications need to occur to confer leukocyte adhesive properties during inflammation? First, large HA “cable-like” structures appear and their formation is dependent on crosslinking of HA with the heavy chain proteins of the serum component, inter-alpha trypsin inhibitor (I*α*I) [8]. The heavy chains of I*α*I, whose attachment to HA had previously been shown in a noncell system to increase adhesiveness of HA for leukocytes [19], were also found to be essential for leukocyte binding ability by cells [8]. HA cable-like structures containing heavy chains of inter-alpha trypsin inhibitor, similar to those produced by colon cells, are now known to be produced by cells of many other tissues, including the kidney [20], lung [21], and vasculature [22] indicating that the process of HA leukocyte recruitment/retention into extravascular tissue is not intestine specific. Importantly, in colon tissue from patients with IBD [8], as well as mice with induced colitis [23], the HA that accumulates in the tissue specifically during inflammation is associated with components of inter-alpha trypsin inhibitor, presumably supplied as serum leaks through the microvasculature during inflammation.

The presence of I*α*I-decorated HA during inflammation raises the question of whether HA is the cause or the consequence of intestinal inflammation. The answer is most clearly demonstrated in the mouse colitis model [9] where intestinal changes in HA deposition were followed over time during the development of inflammation. In this model, animals fed dextran sulfate sodium (DSS) develop breaches in their intestinal epithelial lining allowing intestinal bacteria to cross into the sterile tissue and induce inflammation. Even before the increased presence of leukocytes is observed in the colon, evident changes occur in HA distribution and levels of deposition. One of the earliest changes observed during the course of inflammation in this model is HA presence in the blood vessels of the colon, which is then followed by increased deposition in the submucosa. In the vascular and extravascular tissue of the colon, HA remodeling precedes the infiltration of leukocytes consistent with a role in leukocyte recruitment. Importantly, Kessler et al. [24], in this issue of the journal, demonstrate using the same DSS colitis model, in which mice that have a null deletion of one of the possible HA synthases, HAS3 (but not HAS1), have highly decreased leukocyte infiltration into colon tissue. Whereas the wild type mice eventually show major tissue architecture breakdown, the HAS3 null mice have strikingly less inflammation and less tissue disruption. These results are particularly interesting because HAS3 is regulated by inflammatory cytokines in human endothelial cells derived from the intestinal microvasculature, and the data suggest that microvascular HA may contribute to leukocyte recruitment during colitis.

Several recent studies have also addressed how HA may also be used therapeutically to down regulate inflammation in intestinal disease settings. Work by Zheng and colleagues [25] has shown that intraperitoneal injection of fragmented yet fairly large molecular weight (ave ~0.5 × 10^6^ Da) HA protects mice from damage during induced colitis and this was mediated via TLR4 receptors driving COX2 production that promotes epithelial repair. The cellular mechanism mediating protection is similar to that Misra et al. [26] have previously demonstrated in colonic epithelium* in vitro*. In addition, Riehl and his colleagues [27] have gone on to demonstrate that the protection afforded by intraperitoneal injection of HA provides benefit in radiation induced damage models as well, by sparing the intestinal mucosa. The route of delivery in these therapeutic studies suggests that HA acts to systemically decrease inflammation and promote epithelial repair* in vivo*.

### 2.3. A Role for HA in Intestinal Protection and Antibacterial Defense

Asari et al. [28], using oral delivery of HA around 0.9 × 10^6^ Da, also demonstrated protection of immune compromised mice from inflammation in a TLR4 requiring process. However, in dog and rat models, it has been demonstrated that very little large molecular weight HA is absorbed through the gastrointestinal tract [29]. Therefore, theoretically, because the intestinal epithelium is a barrier to large molecular weight HA, the effect reported may better reflect protection of gut epithelium and prevention of bacterial translocation rather than direct inhibition of inflammatory responses.

Hill et al. [15] recently showed that HA of average molecular weight ~35,000 Da (HA-35), but neither smaller nor larger sized HA, increases epithelial expression of human *β*-defensin 2 (HBD2) protein. This induction also relies on TLR4* in vivo*, as mice lacking the specific receptor were not sensitive to HA-35 induction of the murine orthologue of HBD2 [15]. HBD2 is a naturally produced antimicrobial peptide that has broad-spectrum activity against bacteria, fungus protozoa, and viruses [30]. HBD2 is one of the enteric defensins which is thought to shape the intestinal microflora composition [31], and dysregulation of HBD2 is associated with subtypes of IBD [32]. Therefore, consumption of HA stimulates innate epithelial responses that conceivably could protect the intestine from pathogenic microbes or regulate the homeostatic balance of the intestinal microflora. Low molecular weight HA induction of TLR4-mediated HBD2 production has also been identified in skin [13] and vaginal epithelium [14] suggesting that the HA response is a common epithelial innate response. But, biologically, why would this mechanism exist?

**Figure 3:**
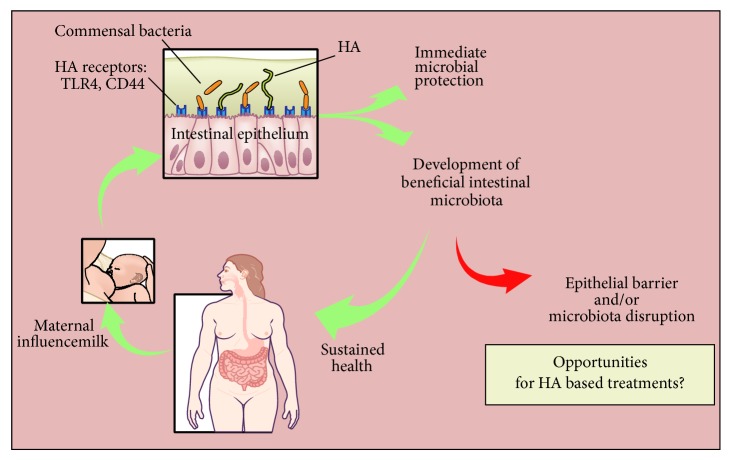
HA in milk may strengthen epithelial defense in the infant gut and foster development of a beneficial microbiota as the baby matures. Appropriate microbial colonization of the intestine is thought to contribute to sustained health into adulthood.

As a partial answer to HA's intestinal role, it was recently demonstrated that HA is a natural component of human milk [33, 34]. Our group has determined that HA is produced at the highest concentrations during the first months after giving birth (~500 ng/mL) and tapers to a steady level (~100 ng/mL) throughout the first year [34]. HA in human milk, therefore, may help protect the mostly sterile newborn gastrointestinal tract from intestinal pathogens and foster colonization with a beneficial microbiota over time (Figure 3). This is consistent with the function of other milk glycans, the human milk oligosaccharides (HMOs) that have been appreciated as beneficial agents that promote healthy commensal bacteria and pathogen protection within the infant gut for over sixty years [35].

Importantly, HA purified from milk, when provided at the physiological concentrations provided to babies, induces increased expression of HBD2 protein by human epithelial cell lines, as well as protection from intracellular* Salmonella typhimurium* infection* in vitro*. Milk derived HA also induces* in vivo* expression of the orthologue of HBD2 in the intestine of mice fed the preparation once daily for three days. However, the activity of milk HA differed from HA-35 in two major ways; in the case of purified milk HA, both TLR4 and a second HA receptor, CD44, are required to induce the HBD2 orthologue* in vivo*. Additionally, the peak effective dose of milk HA began at ~500 ng/mL, a 700-fold lower concentration compared to that required for HA-35 peak activity. Perhaps milk HA is more potent because it engages at least two HA receptors [34]. Examination of molecular mass indicates that less than 5% of milk HA is in the range of HA-35, and 95% is much closer to the effective size [36] described by Asari et al. [28] for intestinal protection.

## 3. Conclusion

The intestine relies on HA not only for homeostatic functions but also in innate responses. Although there are many aspects still to be explored, the best understood responses are in the innate immune context, where HA promotes rapid leukocyte recruitment upon colon tissue damage or bacterial challenge, and fragmented HA stimulates inflammatory cytokine production by mononuclear leukocytes. This role of HA during inflammation, with slightly differing permutations, is common to many other organ systems such as the lung [21], liver [37], kidney [20], and skin [38] too.

Recent data also highlight a less appreciated function for HA, that of promoting epithelial defense mechanisms in the intestine [15, 34]. In this arena, HA has the potential to be a novel dietary supplement for formula fed infants and children at risk for enteric bacterial infection, as well as patients who suffer from bacterial dysbiosis, for example, individuals with inflammatory bowel disease (IBD). Due to growing bacterial antibiotic resistance, there is an increasing call for a larger arsenal of nontraditional antibacterial strategies. We think HA fits in that category and will function to heighten innate defenses.
